# Mobilizing community-led health promotion: Evidence-informed co-development of the Live Well PEI community mobilization platform and integrated granting program

**DOI:** 10.17269/s41997-025-01140-3

**Published:** 2026-01-14

**Authors:** Shamara Baidoobonso, Emily C. Clark, Laura Lee Noonan, Jiselle Bakker, Jennifer L. May-Hadford, Karen A. M. Phillips, Melissa D. Rossiter, Karen B. Kuzmich, Brooks Roche, Travis J. Saunders, David Sabapathy

**Affiliations:** 1 Department of Health and Wellness, Government of Prince Edward Island, Chief Public Health Office, Charlottetown, PE Canada; 2 McMaster University, Hamilton, ON Canada; 3 Public Health Agency of Canada, Regulatory, Operations and Emergency Management Branch, Ottawa, ON Canada; 4 Department of Applied Human Sciences, University of Prince Edward Island, Charlottetown, PE Canada; 5 Canadian Cancer Society, Toronto, ON Canada; 6 Toronto, ON Canada

**Keywords:** Community grant program, Community mobilization, Community engagement, Health promotion, Public health, Programme de subvention communautaire, Mobilisation communautaire, Engagement communautaire, Promotion de la santé, Santé publique

## Abstract

**Intervention:**

Live Well PEI provides individuals and organizations in PEI with information and tools to support healthy behaviours. This paper describes the development of the Live Well PEI community-mobilization intervention, consisting of a website and integrated community granting program for health promotion initiatives.

**Research questions:**

(1) What is an effective design of a web-based community-mobilization intervention for community-led health promotion and (2) what lessons can be learned and shared for community-engaged, evidence-informed public health intervention development?

**Methods:**

Intervention development followed the Six Steps in Quality Intervention Development (6SQuID) process. Through literature reviews, jurisdictional scans, expert consultations, and community engagement, evidence for conditions that foster community mobilization was gathered and used to inform development of intervention components. The intervention was refined through iterative expert and community consultations.

**Results:**

Four conditions that foster community-led health promotion were identified: accessibility of grant funding, availability of community data, resources to enhance capacity, and community collaboration. These conditions informed outputs of a Theory of Change. Intervention design that supports these conditions included website elements such as collections of evidence-informed health promotion interventions, stories of prior community-led projects, and community health status and needs information. The granting program integrates research and community feedback on project eligibility, grant administration and support for awardees. Findings from an evaluability assessment support pilot-testing implementation of the intervention.

**Conclusion:**

Development of the community-mobilization intervention provides a prime case example of evidence-informed, community-engaged public health intervention co-design. Learnings are applicable to community-focused health promotion initiatives across Canada.

## Résumé

**Intervention:**

Mieux vivre Î.-P.-É. fournit aux particuliers et aux organisations de l’Île-du-Prince-Édouard des informations et des outils pour favoriser des comportements sains. L’article décrit l’élaboration de l’intervention de mobilisation communautaire qu’est Mieux vivre Î.-P.-É., qui comprend un site web et un programme intégré de subventions communautaires pour des initiatives de promotion de la santé.

**Questions à l’étude:**

1) Qu’est-ce qu’une conception efficace d’intervention de mobilisation communautaire en ligne pour la promotion de la santé menée par les communautés? 2) Quels enseignements peuvent être tirés et partagés pour l’élaboration d’interventions de santé publique fondées sur des données probantes et impliquant les communautés?

**Méthodes:**

L’élaboration de l’intervention a suivi le processus en six étapes pour l’élaboration d’interventions de qualité (6SQuID). Grâce à des analyses documentaires, des analyses de ce qui se fait ailleurs, des consultations d’experts et la participation des communautés, des données probantes sur les conditions favorables à la mobilisation communautaire ont été recueillies et utilisées pour éclairer l’élaboration des composantes de l’intervention. L’intervention a été affinée grâce à des consultations itératives avec des experts et les communautés.

**Résultats:**

Quatre conditions favorisant la promotion communautaire de la santé ont été établies: l’accessibilité de subventions, la disponibilité de données communautaires, des ressources pour accroître la capacité et la collaboration communautaire. Ces conditions ont étayé les extrants d’une théorie du changement. Le concept d’intervention appuyant ces conditions comprenait des éléments du site Web, comme des interventions de promotion de la santé fondées sur des données probantes, des résumés de projets communautaires antérieurs et des renseignements sur la santé communautaire et les besoins. Le programme de subvention intègre la rétroaction de scientifiques et de communautés sur l’admissibilité des projets, l’administration des subventions, et le soutien aux personnes ou entités subventionnées. Les constatations d’une évaluation de l’évaluabilité appuient la mise en œuvre d’un essai pilote de l’intervention.

**Conclusion:**

La conception de l’intervention de mobilisation communautaire fournit un exemple type de conception d’intervention en santé publique sollicitant les communautés et fondée sur des données probantes. Les leçons tirées s’appliquent à des initiatives de promotion communautaire de la santé partout au Canada.

## Background

Health promotion is a critical function within public health, encompassing interventions that enable individuals and communities to increase control over and achieve health and wellness (World Health Organization, 2021). Community-led initiatives where community groups design and deliver wellness initiatives (e.g., walking groups, nutrition skills, education on alcohol harms), are an essential aspect of health promotion. These initiatives are especially important for reaching communities facing health disparities, i.e., preventable differences in health between groups of people (Haldane et al., 2019; Hancock, 2009). This approach has been shown to improve population health while reducing health disparities. (O'Mara-Eves et al., 2015). Several systematic reviews of published literature support the potential for community-led initiatives in improving health outcomes. Recent systematic reviews have found promising results for health outcome improvement from urban community-led health promotion initiatives. The greatest changes were seen in health promotion-relevant outcomes, such as physical activity and quality of life (Haldane et al., 2019; Nickel & Knesebeck, 2020). A systematic review and meta-analysis focusing on populations made vulnerable through systemic inequities uncovered significant effects on health behaviours, health behaviour self-efficacy and social supports through community engagement in public health interventions (O'Mara-Eves et al., 2015).

Health information is increasingly sought from online sources (Wang et al., 2021), and online platforms are a promising strategy for influencing health behaviours. For diet and physical activity, systematic reviews provide evidence that internet-based interventions can improve outcomes in adults (Lee et al., 2025; Nunns et al., 2025). Similarly, systematic reviews have found that online interventions can help reduce tobacco use (Shi et al., 2024), and may influence alcohol use (McDermott et al., 2023). A trusted, online platform providing health information tailored to the local community was seen as a potential mechanism for improving the health of residents in Prince Edward Island (PEI).

Since 2015, the Health Promotion unit in the Chief Public Health Office (CPHO) of the Government of PEI’s Department of Health and Wellness has sought to support community action in promoting health through the Live Well PEI healthy living initiative (www.livewellpei.ca). The mandate for the online Live Well PEI platform seeks to connect community members and groups with the resources needed to improve health behaviours. The primary goal for Live Well PEI is to reduce chronic disease in PEI by targeting diet, physical activity, tobacco and alcohol use, and mental wellness.

Live Well PEI has several components, including a website and a community granting program ($500-$7,500) available to community organizations, municipalities, Indigenous communities, and schools. The website provides resources to support community members in making healthy behaviour changes, while the community granting program funds community-driven health programs. A first version of the Live Well PEI website was available online to provide educational resources and information. This included general information relevant to health promotion and resources and tools to help support healthy behaviours. Primary target users were the public, and web-based tools were not available to assist community group and organization leaders, staff or volunteers in advancing community health. The CPHO also administered a paper-based community granting program that preceded the Live Well PEI initiative and was not integrated with the design of the Live Well PEI website. Recognizing challenges in supporting community groups’ development and implementation of grant projects, the CPHO sought to redesign its existing community granting program and website as a single evidence-informed, community-informed, community-mobilization intervention. While there is evidence that small grants for community health promotion can engage community organizations and drive innovations for health (Hartwig et al., 2006), there is little guidance for developing effective community granting interventions. The goal of this initiative was to design an effective, evidence-informed web-based community-mobilization intervention for community-led health promotion.

The purpose of this paper is to describe the design and development of the Live Well PEI community-mobilization intervention through iterative consultations of published and unpublished literature, and with members of community-based organizations. The paper address two specific research questions: 1) what is an effective design of a web-based community-mobilization intervention for community-led health promotion, and 2) what lessons can be learned and shared for community-engaged, evidence-informed public health intervention development? This two-year project provides an exemplary case of integrating evidence from research and community to optimize the design and development of a public health intervention and can serve as a guiding example for public health in Canada.

## Methods

### Setting

PEI is Canada’s smallest province, by land area and population. Located in Atlantic Canada, PEI has a population of approximately 180,000, and about 34% of PEI’s residents live in rural areas (PEI Statistics Bureau, 2023).

Public health in PEI is governed by the provincial government’s Department of Health and Wellness. Within this department, the CPHO is responsible for advancing health promotion by developing community interventions that focus on modifiable health behaviours. Given PEI has only one health region, the CPHO’s mandate covers the entire province. Live Well PEI was envisioned as the voice of health promotion in PEI, connecting residents to resources that support improving healthy behaviour change.

Development of the Live Well PEI community-mobilization intervention was funded through the Government of Canada’s Healthy Canadians and Communities Fund (HCCF), with additional financial contributions from the Government of PEI, SPOR Evidence Alliance, and Health PEI. The HCCF supports interventions that address health inequities for chronic disease prevention. Project funding was awarded to support the co-design and co-development of new interventions through engagement of priority populations (Government of Canada 2023).

To support intervention design, project committees were established, consisting of: representatives for the provincial government, the provincial health authority, and academia with expertise in health promotion, evaluation and community engagement; members of community organizations with health promotion activities; individuals with lived experience of chronic disease; and members of populations at greater risk of chronic disease. Committee members provided in-kind contributions and are referred to as project partners in this paper. Additional community groups and organizations and members of populations at greater risk of chronic disease were also consulted at several points during the project.

### Study design

**Fig. 1:**
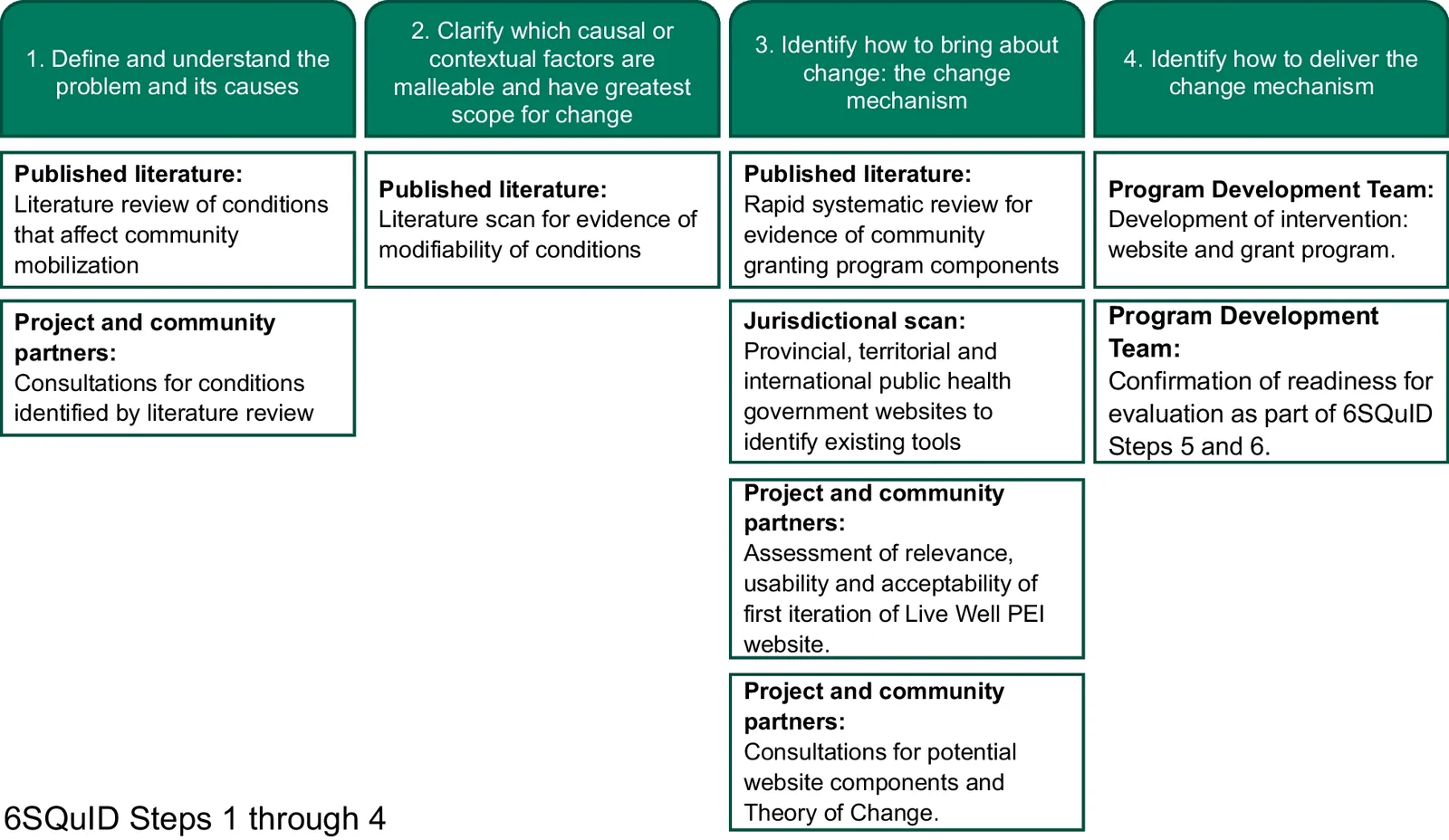
Program development stages, outlining the sources of evidence gathered at stages 1–4 of the 6SQuID framework

The development of the public health intervention followed the Six Steps in Quality Intervention Development (6SQuID) process. The 6SQuID model was developed to provide guidance for logic-based and evidence-informed development of interventions (Wight et al., 2016). The model guides public health researchers or practitioners through six essential steps: 1) defining the problem and understanding its causes; 2) prioritizing which causal factors are modifiable and have the greatest potential impact; 3) choosing an intervention for change; 4) determining how to implement the intervention; 5) testing and adapting the intervention; and 6) collecting pilot data to justify broader implementation and evaluation (Wight et al., 2016). The activities conducted to support development of the community-mobilization intervention are aligned with Steps 1–4 of the 6SquID model, as depicted in Fig. 1 and described below. Step 5 (testing and refining the intervention on a small scale) and step 6 (collecting sufficient evidence of effectiveness to justify full-scale implementation) will be completed through further development of the Live Well PEI platform, and are beyond the scope of this paper.

Intervention design was also influenced by two related models for health behaviour: the Theory of Triadic Influence and the Socio-Ecological Model (Flay et al., 2009; Scholmerich & Kawachi, 2016). The Theory of Triadic Influence posits that the ultimate, upstream causes of health-related behaviour can be categorized as the cultural environment, the social situation, and biology or personality. The Socio-Ecological Model similarly attributes health behaviours to societal, community, and relationship-level influences. Together, these models support the understanding that interventions at the community level can influence health behaviours, since they influence the context for the behaviour beyond the individual.

### Data collection and analysis

A multifaceted approach was taken to collecting data and evidence from published literature, unpublished literature, and project and community partners to support co-development of the community-mobilization intervention. Collection and analysis of data was conducted iteratively, where the data collected in one step of the 6SQuID process was analysed, interpreted, and used to inform a future step. This subsection therefore describes the collection and subsequent analysis of data within steps of the 6SQuID process (Fig. 1).

In the first step of the 6SQuID process, conditions that facilitate community mobilization were identified in published literature, then feedback was gathered from project partners and community members. In the second step, evidence was gathered for the modifiability of these conditions, that is, the extent to which they can be changed by intervention. In the third step, website and grant program components with the potential to support the conditions were identified in published and grey literature, and feedback on a draft Theory of Change was gathered from project and community partners. In the fourth step, the intervention was designed and its readiness for evaluation was assessed. These activities are described in further detail below.

#### Literature review of conditions that affect community mobilization (6SQuID Step 1)

To inform the prioritization of elements to include in the intervention, a non-systematic literature review was conducted to identify specific conditions that impact the capacity for community groups or organizations to engage in health promotion activities. This literature review was conducted by a single reviewer searching PubMed, Medline and CINAHL databases. Qualitative studies, case studies, scoping reviews, systematic reviews and editorials that explored the implementation of community-based health promotion, including lessons learned, common practices within the field, and recommended best practices, were reviewed. The reviewer assessed the relevance of each articles, extracted information about impactful conditions, and grouped related conditions into categories.

#### Consultations for conditions identified by literature review (6SQuID Step 1)

The list of conditions that support community mobilization were shared with project partners during a hybrid in-person/virtual consultation. The objective of the consultation was to engage project partners in validating the information gleaned from the literature review and identifying conditions to prioritize for intervention development. In small groups, participants discussed which conditions were relevant to their organizations, additional conditions not captured in the literature review, and modifications required to adapt conditions to the local PEI context.

Prioritized conditions for community mobilization were also shared with community members during an in-person consultation session. Community members shared their reflections on which conditions they felt would support community mobilization based on their lived experiences.

#### Evidence for modifiability of conditions (6SQuID Step 2)

In the second step of the 6SQuID process, evidence was collected to inform the potential of the community-mobilization intervention to affect prioritized conditions. Published literature was scanned for interventions with demonstrated effectiveness for establishing or enhancing conditions. Article screening and data extraction were conducted independently using a shared template; grouping themes was done collaboratively amongst the review team.

#### Rapid systematic review of community granting program components (6SQuID Step 3)

To inform the components of a community granting program that would support meeting conditions for community mobilization, a rapid systematic review was conducted by the National Collaborating Centre for Methods and Tools’ Rapid Evidence Service (Clark et al., 2024; Neil-Sztramko et al., 2021). The methodology for this rapid systematic review is published in detail elsewhere (Clark et al., 2024).

#### Jurisdictional scan of provincial, territorial and international public health government websites to identify existing web-based tools (6SQuID Step 3)

To explore existing web-based tools that support community-led health promotion, a jurisdictional scan of available tools, resources and services was conducted. Interactive tools designed to drive community action were included. The scan surveyed both provincial and territorial public health websites in Canada, as well as regional and national public health websites in Australia, Finland, Hungary, Ireland, New Zealand, Switzerland, Sweden, the United Kingdom, and the United States. Information regarding the components of the tool (e.g., webpage, community health indicators map, technical support, etc.) was extracted.

#### Consultations for feedback on the first iteration of Live Well PEI website (6SQuID Step 3)

Feedback was solicited on the first version of the Live Well PEI website through focus groups and interviews with participants recruited through community groups and organizations and through networks of project partners. Discussion questions focused on the website’s relevance, ease of use and areas of improvement.

#### Consultations for potential website and granting program components and Theory of Change (6SQuID Step 3)

Project partners were also asked to rate potential website elements for relevance, feasibility and importance and potential impacts. Guiding questions to support ratings are included in the Appendix. Ratings were determined through discussion until consensus was achieved.

Additional consultations were held with project partners and community members. The objectives of these consultations were twofold: 1) for project partners to further co-develop the intervention’s Theory of Change and Logic Model, and 2) to gather feedback from project partners and community members on potential website components and grant program criteria.

#### Design of intervention: Website and grant program (6SQuID Step 4)

Information gathered and analyses completed in Steps 2 and 3 were synthesized to identify website elements that supported conditions for community mobilization and rated highly across all domains.

Storyboards were designed around each of these elements and used in stakeholder consultations with project partners, community members, and information technology and web development specialists. Following the consultations, the storyboards were revised and then translated into technical and business requirements that formed the basis of the final website design.

As with the website, the structure for the grant program was redesigned based on information from Steps 2 and 3, a storyboard and feedback from project partners and community members. All facets of the granting program, including grant project eligibility criteria, grant program administration, program structure and supports were analyzed. Each option was reviewed for supporting evidence; for example, whether there was evidence in the review of published literature or comments from project or community partners. The feasibility of each option was considered by the project team and CPHO management in making final selections.

#### Evaluability assessment (6SQuID Step 4)

To inform 6SQuID Steps 5 and 6, an evaluability assessment was conducted to ensure readiness for evaluation that would co-occur with implementation of the website and grant program. The evaluability assessment was conducted through focused sessions with the project team and document review. It assessed the project team’s understanding of the intervention, commitment to utilizing evidence collected during intervention design, the Theory of Change underlying the intervention and availability of necessary data. Findings were interpreted in in the context of the RE-AIM framework, which assesses interventions according to Reach, Effectiveness, Adoption, Implementation and Maintenance (Glasgow et al., 1999).

## Results

Findings were generated during each stage of the 6SQuID process. These include the conditions that foster community-led health promotion activities in Steps 1 and 2, the Theory of Change and Logic Model in Step 3, the online platform elements and grant program design in Step 4, and the formative evaluation of the intervention to inform its design and implementation, thereby contributing to Steps 5 and 6. These findings are presented in detail below.

### Conditions that foster community-led health promotion activities

Searching for the non-systematic literature review retrieved 788 articles, of which 16 were included in the review. Four conditions that foster community mobilization for health promotion were identified through the literature review and consultation with project partners and community members. These include accessibility of grant funding, availability of community data, resources to enhance knowledge and skill, and fostering community collaboration. The criteria for meeting each of these conditions, and supporting evidence for the modifiability of these conditions, are further described below and summarized in Table 1.

1. *Accessibility of funding*: Funding for health promotion activities was identified as an enabling condition for community mobilization; specifically, the availability of grant funding to support project development, implementation and evaluation. This condition was determined to be malleable given various grant models in the published literature that support community action for chronic disease prevention (Cheadle et al., 2016). Grant funding eligibility and priorities should be informed by community. Examples in published literature support community-informed granting programs and involving community members on grant application review committees (Ramos et al., 2013). Finally, the administrative burden to apply for and hold grant funds must be reasonable and accessible within the varying skillsets, resources and time demands of community members. This includes ensuring that the grant application process is clear, the time demands for an application are proportional to the funds offered, and that any project reporting requirements are considerate of community organizations’ time (Cheadle et al., 2016).
2. *Availability of community data*: To inform community-led health promotion initiatives, community health status data should be available in a format accessible to community organizations. Ideally, this data should be stratified by neighbourhood and population characteristics that affect health equity. Examples from the literature support the availability of data allowing for the assessment and prioritization of community needs which enhances grant project proposals (Easterling et al., 2012). Additionally, the experienced successes and challenges of completed projects should be shared with prospective applicants and grant awardees. A process for sharing reflections on project implementation and impact should be established. Literature supports the sharing of best practices and community experiences to build knowledge and community capacity (Cheadle et al., 2016).
3. *Resources to enhance knowledge and skill:* Community organizations need the knowledge to find evidence-based projects that can be implemented in their communities, as well as the skills for implementing those projects (Alexander et al., 2020). To support project planning, implementation and evaluation, community organizations should have access to experts who can support these activities. Examples in the literature demonstrate that technical assistance from experts can increase the capacity of the community to lead health promotion initiatives (Easterling et al., 2012).
4. *Fostering of community collaboration*: To enhance the ability of community organizations to deliver health promotion initiatives, they should be aware of supporting resources and services in their communities. Evidence supports the role of community resources in community-led initiatives (Mosavel et al., 2018). Opportunities for community organizations to network and share learnings and resources should be integrated. These allow for community organizations to identify project synergies, enabling connection and collaboration. Evidence supports knowledge sharing to enhance the effectiveness of initiatives (Barker et al., 2022; McKinnon et al., 2022).

**Table 1:** Conditions that support community mobilization for health promotion activities

Condition	Condition criteria	Modifiability examples (i.e., literature-derived examples of how modification changed environment to improve capacity)
1. Accessibility of funding	• Community projects are influenced by the funding model, including amount of money, timing of money, and the objectives of the fund • Grants should support project development, implementation, and evaluation	Various grant models demonstrate good ability to support community action for chronic disease prevention. These include small microgrants supporting needs assessments, and multi-year grants supporting interventions (Cheadle et al., 2016)
	• Community input is encouraged and considered in developing grant program funding priorities	Understanding local realities and having relationships with communities is needed to set grant priorities and inform successful proposals. Community membership may sit on grant review committees (Ramos et al., 2013)
	• Administrative burden to apply for and hold grant funds is reasonable. Application process is clearly outlined, including key project activities and budget • Time to complete application is reasonable in length and proportional to the amount of funding being offered • Reporting requirements are respectful of the time of community organizations	Evaluations, including community feedback, of grant processes provides useful information for improving grant process Making previous grant applications available increases transparency and fosters learning. Engaging community members as grant reviewers improves grant writing skills (Cheadle et al., 2016)
2. Availability of community data	• Communities have access to local data to support needs assessment and prioritization (e.g., smoking rates, chronic disease rates) • Communities have access to local data that, where possible, is stratified by neighbourhood and characteristics that affect health equity to identify differences between groups	Community health profiles inform decision-making and enhance grant proposal strength. Data for community- specific indicators allows priorities to be explored. Indicators allow comparison between communities and goals/standards (e.g., national standards) (Easterling et al., 2012)
	• Learnings from community projects are shared. Review and reflection on project implementation and impacts allow for understanding of successes and challenges. A process for sharing these learnings should be established	Bulletins provided an opportunity to synthesize knowledge and to value community voices/experiences, while informing future programs. Health promotion experts ensured that evaluations were used to adapt program delivery. Agencies learned from one another. Grant programs that required evaluation at various points during project implementation allowed for early program refinements (Cheadle et al., 2016)
3. Resources to enhance knowledge and skill	• Community organizations have knowledge for health promotion and chronic disease prevention • Community members and organizations can find evidence-based projects for health promotion • Community members and organizations have the skills to implement projects	Utilizing Community Based Participatory Research (CBPR) enhances adoption of context relevant interventions. Collaboratively developed, specifically tailored knowledge tools to disseminate information to other organizations (Alexander et al., 2020). Purposeful investment in knowledge dissemination is required to maximize uptake and benefit (Easterling et al., 2012)
	• Community members and organizations have access to professionals who can share knowledge and resources on developing, implementing, and evaluating community projects (e.g., Health Promoters or Epidemiologists)	Technical assistance including professional development, coaching, and mentoring, consultation, knowledge brokering increases community capacity and is dose dependent. Technical assistance such as training and educating stakeholders, support to design interventions, developing stakeholder relationships, intervention adaptation increases community capacity (Easterling et al., 2012)
4. Fostering of community collaboration	• Community organizations are aware of existing community resources that could support health promotion initiatives	Identifying different services and expertise in a community, such as via community asset mapping, demonstrated good utility to inform and catalyze community project (Mosavel et al., 2018)
	• Sharing of best practices, lessons learned, and resources occurs among staff and volunteers to build capacity and enhance success • Communities able to identify project synergies, enabling connection and collaboration	Collaboration between organizations implementing similar projects allowed lessons learned to be shared, enhancing the quality of programs (McKinnon et al., 2022)
	• Community organizations partner with other organizations to promote health • Projects’ effectiveness increased through drawing on the expertise of other organizations. Different perspectives improve programing effectiveness • Engaging other organizations increases available resources and improves connections to community	Formal partnerships allow access to specialized resources (e.g., psychiatry services in community) and opportunity to expand services into sites serving equity deserving populations. Partnering with community organizations can provide access to volunteer resources increasing sustainability of interventions (Barker et al., 2022)

### Theory of change

Following the identification and prioritization of factors that support community mobilization, the project team co-developed a Theory of Change for the website and granting program, in consultation with project partners. The Theory of Change identifies causal relationships between the CPHO and community’s interaction with the website and granting program, and these interactions’ immediate, short and long-term outcomes.

**Fig. 2:**
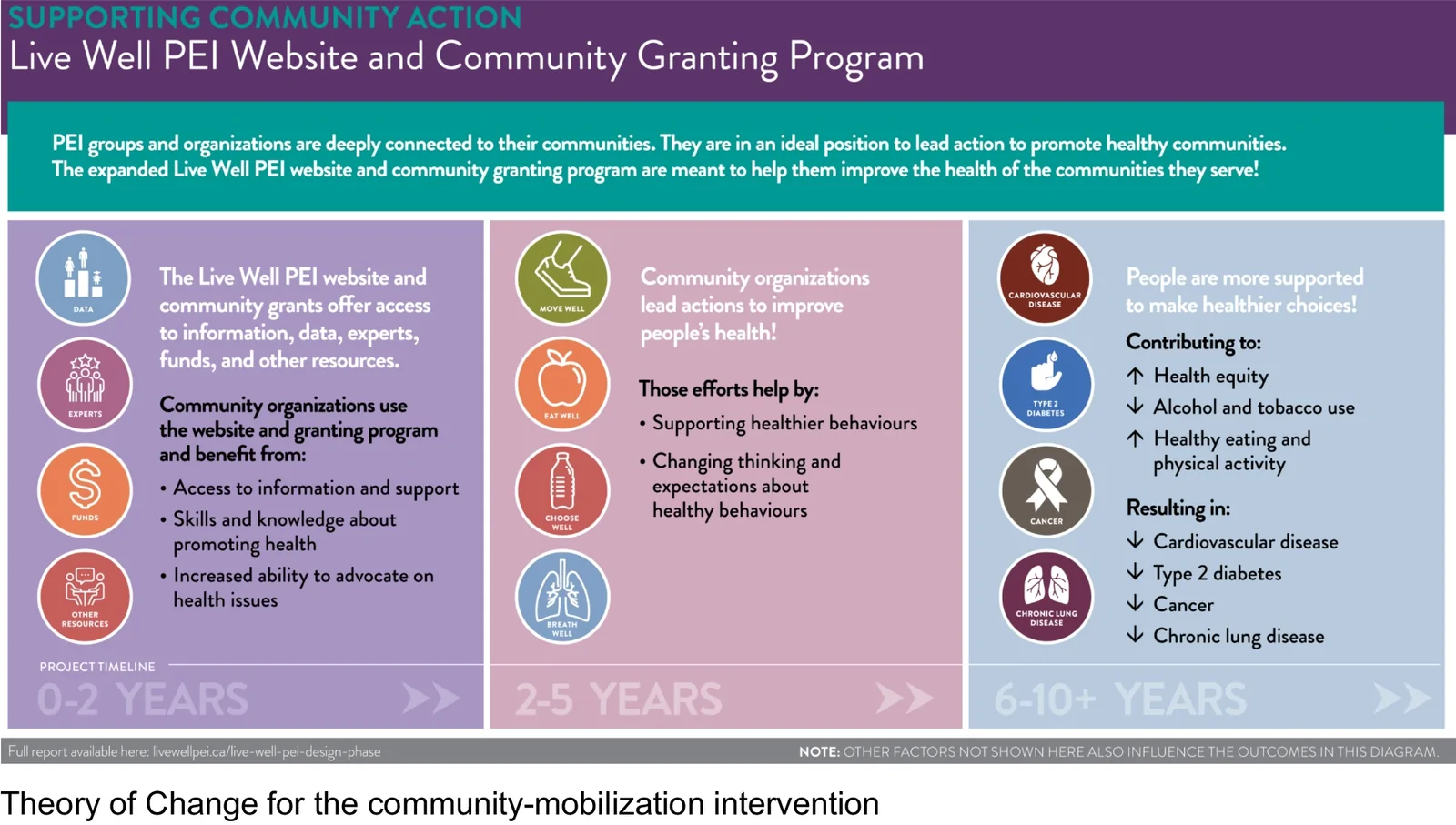
Theory of Change

The Theory of Change is shown in Fig. 2. The website will provide access to community data, evidence-informed interventions, technical expertise, resources, and the integrated community granting program. The immediate outputs of the website and integrated granting program align with the conditions that support community mobilization: community groups and organizations have access to community data, evidence-informed interventions, technical expertise, and resources to build knowledge and skill; and they have increased ability to act on health issues. With access to the outputs of the intervention, the main outcomes of the intervention are community-led, evidence-informed health promotion initiatives. These initiatives will be driven by the community, using funding available through the granting program. Finally, the projected impact of these initiatives are changes to the socio-cultural environment and context to support healthier behaviours, changes to knowledge, skills and self-efficacy to support health behaviours, and ultimately changes to key indicators for chronic diseases and health equity for PEI residents.

### Intervention design: website and grant program

Twenty-one potential website elements were identified through published and grey literature, and project and community partners, and are listed in Table 2. Of these, seven elements were selected for inclusion on the Live Well PEI website. These potential website elements were mapped to the conditions that support community mobilization. Two elements were focused specifically on providing access to community health status information: data visualization tools and information about community needs. Both elements were rated high for relevance, feasibility and importance. Supporting community organizations’ knowledge for health promotion and chronic disease prevention, a collection of evidence-informed health promotion interventions was rated highly by project and community partners. Similarly, a collection of stories about previously funded community-led projects was identified as supporting shared learnings from community projects and rated highly. An online grant management system was considered highly relevant, but the feasibility was rated as unclear, as the resources required for web development of such a system had not yet been estimated. A health promotion-focused newsletter or bulletin was also rated highly for relevance but had mixed ratings from project and community partners for feasibility. This was because the feasibility would be highly dependent on the frequency and content of these newsletters or bulletins. Potential impact was also rated high, given that the possible content could be tailored to support multiple conditions for community mobilization.

**Table 2:** Potential website elements mapped to conditions for community mobilization and rated by project and community partners

Potential website element	Applicable condition criteria for community mobilization	Relevance	Feasibility	Importance & Potential Impact	Decision
Collection of evidence-informed health promotion interventions	Community organizations have knowledge for health promotion and chronic disease prevention	High	High	High	Include
Collection of stories about previously funded community-led projects	Learnings from community projects are shared	High	High	High	Include
Online registration for learning opportunities	Resources to enhance knowledge and skill	High	High	High	Include
Online grant management system	Grant funding is available for community-led projects	High	Unclear	Not rated	Include
Open data portal (i.e., access to local health reports, data tables and datasets)	Community health status data is accessible	High	High	High	Include
Information about community needs	Community health status data is accessible	High	High	High	Include
Health promotion-focused newsletters or bulletins (posted online and/or sent directly to subscribers by e-mail)	Community health status data is accessible; learnings from community projects are shared; community organizations have knowledge for health promotion and chronic disease prevention; community organizations share knowledge, skills, and opportunities	High	Mixed	High	Include
Interactive map to visualize local health data	Community health status data is accessible	High	Medium	Low to med	Exclude
Calendar of community health promotion events	Community organizations share knowledge, skills, and opportunities	High	Low	Low	Exclude
Personal account	N/A (Administrative function)	High	Low	Low	Exclude
Database of community volunteers	Community organizations are aware of existing community resources	Low (already exists in community)	High	Not rated	Exclude
Access to Health Promoters or Epidemiologists via web-based chat	Community organizations have access to experts	Low	Low	Not rated	Exclude
Profiles of community groups and organizations	Community organizations are aware of existing community resources; community organizations collaborate	Low	Low	Not rated	Exclude
Moderated online discussion forum	Learnings from community projects are shared; community organizations share knowledge, skills and opportunities; community organizations collaborate	Low	Not rated	Not rated	Exclude
Links to community resources	Community organizations are aware of existing community resources	Low (already exists in community)	High	Not rated	Exclude
Information about the CPHO Wellness Grant program: structure, process, criteria	Administrative burden to apply for and hold grant funds is reasonable	Low (available on existing website)	Not rated	Not rated	Exclude
Mechanism to provide feedback to CPHO staff	Grant funding eligibility and priorities are informed by the community	Low (available on existing website)	Not rated	Not rated	Exclude
Online courses provided beyond the CPHO's offerings	Community organizations have knowledge for health promotion and chronic disease prevention; community organizations have access to experts	Low (available on existing website)	Not rated	Not rated	Exclude
Form to enable community groups to submit local health promotion content for the Live Well PEI website	Communities able to identify project synergies, enabling connection and collaboration	Low (available on existing website)	Not rated	Not rated	Exclude
Tools and resources webpage	Community organizations have knowledge for health promotion and chronic disease prevention; knowledge and awareness of community resources such as facilities or expertise that could support health promotion initiatives	Low (available on existing website)	Not rated	Not rated	Exclude
Access to Health Promoters or Epidemiologists via a contact form	Community members and organizations have access to professionals who can share knowledge and resources on developing, implementing, and evaluating community projects	Low (available on existing website)	Not rated	Not rated	Exclude

Elements that were rated low for any domain were excluded. This included an interactive map of local health data, since this data was available in other formats. A calendar of community events was rated as low feasibility, given the maintenance required by CPHO staff. A database of community volunteers and links to community resources were rated as low relevance, as discussion with project and community partners revealed that these resources already exist for the PEI community.

A similar approach was taken with designing the new community granting program. Options related to grant project eligibility, grant program administration, and program components are summarized in Table 3, along with evidence from literature and community partners supporting each option. Considering the supporting evidence for each option, as well as the perceived feasibility, project staff selected options for each element of the granting program. Selected design recommendations are listed below.

**Table 3:** Granting program options, supporting evidence and decisions

**Category**	**Options**	**Supporting evidence from literature (** Clark et al., 2024**), project partners and community partners**	**Decision**
**Grant Project Eligibility**	**Grant project area of focus**		
	Specific public health topic area (e.g., health promotion, cancer prevention)	Literature: 71% of community granting programs focused on specific public health topic area; narrow focus was more common among smaller grants	Grant proposals that address a specific public health topic area (e.g., health promotion, cancer prevention) are eligible
	Broad applicability to community health	Literature: 29% of the community granting programs identified had no specific public health topic area; grantees could submit projects for anything related to community health	
	**Eligible applicants**		
	Non-governmental organizations, educational institutions, and governments	Literature: description of “non-governmental organizations, educational institutions, and governments”	Non-governmental organizations, educational institutions, and governments and informal community groups are eligible
	Informal community groups	Project team suggestion to better fit local context	
	**Eligible projects**		
	Communities of practice	Community partners: Funding for communities of practice facilitate learning about various topics	Projects to develop communities of practice and projects in different phases are eligible
	Grants that support different project phases	Community partners: General support for this option. Variation in the types of grants offered (e.g., supporting different levels of programming to meet different organizational capacities and needs). Templates and examples of prior grants would be helpful	
	**Requirement that project proposals are evidence-based**		
	Projects are not required to be based on research evidence	Literature: 80% of the community granting programs did not require project proposals based on research evidence	Proposals must address a priority need identified through community data; they are not required to be research-based
	Project must address a priority need identified through community data	Literature: 6% of the community granting programs required that project proposals address a priority need identified through community data	
**Grant Program Administration**	**Required Letter of Intent prior to Grant Application**		
	Required Letter of Intent prior to grant application	Literature: 11% of the community granting programs identified indicated that projects must submit a letter of intent	A pre-screening form must be submitted and approved prior to full grant application
	Required pre-screening form and approval prior to grant application	Community partners: Pre-screening form may streamline application process	
	**Application format**		
	Application template or form	Community partners: Forms and templates make applications easier to complete. This may be especially beneficial for smaller organizations	An application template or form will be used
	List of application elements	Literature: common application elements include statement of purpose, statement of community need, description of the project or project work plan, the potential impact or description of how the project addresses community needs, list of partners and their roles, the team’s experience and capacity to implement the project, anticipated health outcomes, timeline, evaluation plan, budget with justification	
	**Application review process**		
	Formal rubric or scoring system for each application component	Literature: 29% of the community granting programs used a formal rubric or scoring system to assess applications	Application review will include interviews or presentations. Academics and community reviewers will be included
	Written application and applicant interviews	Literature: 3% of the community granting programs required a written application and interviews	
	Written application and presentation by applicants	Literature: 3% of the community granting programs required a written application and presentation	
	Program leadership or staff review applications	Literature: Program staff and leadership reviewed applications in 26% of community granting programs identified	
	Include academic and community reviewers	Literature: 23% of the community granting programs had academic and community reviewers For community-research partnerships, review committees consisted of both community and research representatives -Two of the programs identified recruited community members to review applications, including one program where community members selected a short list of applications that were then voted on by the public for final selection	
	Provide feedback to nonfunded applications	Literature: 9% of the community granting programs identified provided feedback to non-funded applications and encouraged applicants to revise their applications and reapply in future years	
	**Reporting requirements**		
	Require interim report(s)	Literature: 43% of the community granting programs required grantees to submit at least one interim report for their project. Mid-project updates were often an opportunity for awardees to express their needs for assistance or support from program staff	Projects will be required to submit a final report. The program will convene awardees to present completed projects
	Require a final project report	Literature: 46% of the community granting programs required grantees to submit a final report	
	Convene all grantees at an event to present their completed projects	Literature: 9% of the community granting programs convened awardees to present completed projects	
**Program components**	**Capacity building for awardees**		
	Any technical assistance	Literature: 86% of community granting programs provided technical assistance to grantees Technical assistance was typically provided by program staff and addressed various needs and challenges, such as consultations for application development, guidance for program planning and implementation, or resources to support evaluation	Technical assistance will be available for project planning, implementation, and evaluation. Workshops and training will be provided
	Technical assistance for application development	Literature: 21% of community granting programs allocated program staff to assist grantees with application development. Some programs held scheduled information sessions about the program and application process, while others provided support to applicants on an ad hoc basis	
	Technical assistance for project planning, implementation, and evaluation	Literature and Community partners: 40% of community granting programs provided program staff to assist grantees with all stages of project planning, implementation and evaluation, either through regularly scheduled meetings, on an ad hoc basis, or both. One program described assigning staff to projects as dedicated technical support. Four programs sent program staff to project sites to conduct on-site visits and provide technical support	
	Workshops and training	Literature: Neary half of community granting programs provided workshops and training to awardees, to build knowledge of important concepts and skills for health promotion. Topics also included policy and advocacy, project implementation, etc. Workshops were often opportunities for program staff to connect with awardees, and for awardees to network with one another	
	Mentorship and/or guidance	Literature: 43% of community granting programs provided mentorship or guidance from program staff, academic partners or experts	
	Facilitate conferencing between awardees	Literature: 17% of community granting programs facilitated conferencing for awardees	
	**Grant size**		
	Eliminate smaller awards	Community partners: Greater value in investing in larger projects than offering smaller grants. Securing small amounts of funding may not be justify time spent applying for funds	Smaller awards will be eliminated
	**Grant duration**		
	Multi-year grants	Community partners: High level of support for multi-year grants. Multi-year grants may support maintaining momentum for community projects. There was some concern that projects would lose momentum over longer time periods. More time can be allocated to planning to ensure that funds are well spent	One-year and multi-year grants will be available
	Mix of one-year and multi-year grants	Suggestion from project team	
	**Project evaluation**		
	Provide technical assistance for evaluation	Community partners: Interest in learning about evaluation	Evaluation of projects will be required. Technical assistance for evaluation will be provided. The CPHO will create infographics for completed project evaluations
	Create infographic summaries of evaluations of funded projects	Community partners: Infographic summaries of evaluation may support learning for other organizations. Suggestion that the CPHO creates infographics or infographic templates	

Grant project eligibility.

- Proposals that address a specific public health topic area (e.g., health promotion, cancer prevention).
- Proposals that address a priority need identified through community data; they are not required to be research-based.
- Projects that develop communities of practice and projects in different phases.
- Non-governmental organizations, educational institutions, governments, and informal community groups.

Grant program administration

- A pre-screening form submitted and approved prior to full grant application.
- An application template or form.
- Application review includes interviews or presentations. Reviewers include academics and community members.
- Project teams are required to submit a final report.
- The program convenes awardees to share completed projects.

Program components

- Technical assistance available for project planning, implementation, and evaluation. Workshops and training provided.
- One-year and multi-year grants available.
- Evaluation of projects required. Technical assistance for evaluation and CPHO-created infographics of completed project evaluations.

Together, these grant program design options will inform the redesign and implementation of the community granting program component of the community-mobilization intervention.

### Evaluability

The evaluability assessment found readiness for evaluation of the intervention both during and after implementation. The Theory of Change and Logic Model were seen as clear and providing a theoretical basis linking the intervention to the intended outcomes (see Fig. 2). Project team members had a shared understanding of the intervention, had clearly defined informational needs for evaluating the intervention once it is implemented, and are committed to evaluating the intervention. Evaluation efforts were planned in alignment with the RE-AIM framework:

- *Reach*: Review of key web usage indicators for webpages intended for community organizations and groups, collected pre- and post-implementation.
- *Effectiveness* and *Adoption*: Analysis of data from surveys and interviews with community groups and organizations, as well as assessment of quality of submitted grant applications to gauge uptake and influence of intervention resources.
- *Implementation*: Comparative analysis of the community-mobilization intervention and its design elements.
- *Maintenance*: Analysis of interviews conducted with managers for the Health Promotion and Population Health Assessment and Surveillance units in the CPHO.

A longer-term evaluation of the intervention’s impact on rates of chronic disease and related health behaviours is planned for 6–10 years following initiation of the intervention. The evaluability assessment and plans for evaluation confirmed readiness for evaluation in 6SQuID steps 5 and 6.

## Discussion

The development of the Live Well PEI website and integrated community granting program provides a rich example of rigorous, evidence-informed, community-engaged public health intervention co-development. The intervention is strengthened by following the 6SQuID process for public health interventions, conducting systematic and thorough reviews of published and grey literature, and engaging community members at every stage of development. This paper describes evidence-based processes used to design intervention components to foster community mobilization for community-based health promotion.

The conditions that foster community mobilization were critical in the design of this intervention. These informed the outputs of the intervention, as depicted in the Theory of Change, ensuring the intervention components aligned with the intended change mechanism. Mapping intervention components, such as website elements and grant program components to these conditions ensured the final design met the objectives of the intervention. For example, one key condition was that resources to enhance knowledge and skills are made available to community organizations. This was integrated into the website design by including a list of learning resources that support project planning, implementation and evaluation, as well as contact information to receive support from members of the CPHO health promotion team. This process reflected the importance of Steps 1 and 2 of the 6SQuID process for public health intervention development and supports its broad application for public health in Canada.

Development of this intervention took a multi-faceted approach to evidence collection, integrating evidence from research, community members, and feasibility through the project team’s public health expertise. This provides a case example operationalizing the model for evidence-informed decision making in public health (National Collaborating Centre for Methods & Tools, 2022). The various sources of evidence were consulted at several stages of intervention development in a highly iterative process, supporting the dynamic nature of this model and its applicability to complex public health interventions.

Despite PEI’s small population and public health workforce, this project was evidence-informed and thoughtfully designed, involving diverse partners. This allows for broad relevance and applicability to other jurisdictions. The project’s iterative approach of consulting community members at various stages of development provides a tangible example of co-production in public health which leads to high community engagement (Vargas et al., 2022). Public health organizations seeking to involve community members in project planning can adapt Live Well PEI’s approach to engaging community members and integrating feedback and multiple stages of design. While there are examples of co-production for specific health promotion initiatives in the literature, to the authors’ knowledge, Live Well PEI provides a novel contribution in describing co-production of a broader initiative that supports community members and organizations in developing their own health promotion projects (Clark et al., 2024).

The next steps for the Live Well PEI intervention will include pilot testing (6SQuID Step 5) and collecting evidence of effectiveness to support full-scale implementation (6SQuID Step 6).of the new elements of the website and redesigned community granting program. Evaluability of the intervention has been confirmed, and the CPHO will capture baseline data and evaluate the intervention to assess implementation and capture outcomes for community mobilization. Upon implementation of the full Live Well PEI platform, residents and community organizations in PEI will have greater resources and opportunities to shape health promotion in the province. Ongoing evaluation of the platform, community wellness initiatives, and population health indicators is anticipated to support sustained investment in community-led health promotion.

**Contributions to knowledge.**

What does this study add to existing knowledge?

- This study informs conditions required for community mobilization for health promotion initiatives. The conditions identified through this study are transferrable for other community-mobilization interventions in other settings and contexts.
- This study also provides key elements of interventions to support community mobilization that can be adapted to other contexts.

What are the key implications for public health interventions, practice, or policy?

- This study provides an exemplar for rigorous, evidence-informed, community-engaged development of public health interventions in the Canadian context.
- This study also demonstrates the capacity of a smaller organization (i.e., PEI’s CPHO) to engage in intervention design.
- Design of public health interventions requires iterative consultation of various sources of evidence, including research and unpublished literature, experts and community partners to optimize potential impact on community health behaviours. The design process was enhanced by using existing models and theories, e.g., 6SQuID, RE-AIM, to guide design.

## Data Availability

All data generated are available upon reasonable request.

## Appendix

Criteria for assessment of proposed tools, resources services (features)

**Table:**

Domain	Question/concept to guide assessment
**Relevance**	To which domain of the Theory of Triadic Influence does tool, resource, or service map?
	Is there evidence from research, evaluation, or theory that this tool, resource or service works?
	Does this tool, resource or service currently exist for PEI community groups and organizations?
	Does this tool, resource or service align with health promotion strategies and actions as defined by the Ottawa Charter? (World Health Organization, 1986)
**Feasibility**	What are some of the non-technical, non-cost implementation considerations (e.g. associated activities that may be required, barriers, facilitators, sustainability/maintenance) associated with this tool, resource or service?
	How technically difficult and suitable would it be to add this tool, resource, or service to the Live Well PEI website?
	What is the cost (range) of technical and implementation requirements for this tool, resource or service?
**Importance and Impacts**	Impressions of community groups/organizations (from Community Workshop)
	What is the potential benefit of this tool, resource or service by itself? What is the level of certainty for each benefit?
	Are there any potential drawbacks related to this tool, resource or service? What is the level of certainty for each drawback? Consider harms, which can be direct, psychological, equity-related, group/social, and opportunity, etc
	Does this tool, resource or service impact/influence or is impacted or influenced by other tools, resources or services on this list? Which ones?
	Are there any equity considerations associated with this tool, resource or service? If so, what are they?

## Abbreviations

*6SQuID*: 6 Steps in Quality Intervention Development
*CPHO*: Chief Public Health Office
*HCCF*: Healthy Canadians and Communities Fund
*PEI*: Prince Edward Island
